# The causality between CD8^+^NKT cells and CD16^−^CD56 on NK cells with hepatocellular carcinoma: a Mendelian randomization study

**DOI:** 10.1186/s13027-024-00565-8

**Published:** 2024-01-20

**Authors:** Zhengmei Lu, Xiaowei Chai, Yong Pan, Shibo Li

**Affiliations:** 1 Department of Infectious Diseases, Zhoushan Hospital, Wenzhou Medical University, Zhoushan, 316021 China; 2 Tongji Hospital Affiliated to Tongji University, Shanghai, 200040 China

**Keywords:** Hepatocellular carcinoma (HCC), Mendelian randomization (MR), CD8^+^NKT cells, CD16^−^CD56 on NK cells

## Abstract

**Background:**

Hepatocellular carcinoma (HCC), which is featured with high morbidity and mortality worldwide, is a primary malignant tumor of the liver. Recently, there is a wealth of supporting evidence revealing that NK cell-related immune traits are strongly associated with the development of HCC, but the causality between them has not been proven.

**Methods:**

Two-sample Mendelian randomization (MR) study was performed to probe the causal correlation between NK cell-related immune traits and HCC. Genetic variations in NK cell-related immune traits were extracted from recent genome-wide association studies (GWAS) of individuals with European blood lineage. HCC data were derived from the UK Biobank Consortium's GWAS summary count data, including a total of 372,184 female and male subjects, with 168 cases and 372,016 controls, all of whom are of European ancestry. Sensitivity analysis was mainly used for heterogeneity and pleiotropy testing.

**Results:**

Our research indicated the causality between NK cell-related immune traits and HCC. Importantly, CD8^+^NKT cells had protective causal effects on HCC (OR = 0.9996;95%CI,0.9993–0.9999; *P* = 0.0489). CD16^−^CD56 caused similar effects on NK cells (OR = 0.9997;95%CI,0.9996–0.9999; *P* = 0.0117) as CD8^+^NKT cells. Intercepts from Egger showed no pleiotropy and confounding factors. Furthermore, insufficient evidence was found to support the existence of heterogeneity by Cochran's Q test.

**Conclusion:**

MR analysis suggested that low CD8^+^NKT cells and CD16^−^CD56 expression on NK cells were linked with a higher risk of HCC.

**Supplementary Information:**

The online version contains supplementary material available at 10.1186/s13027-024-00565-8.

## Introduction

**Fig. 1:**
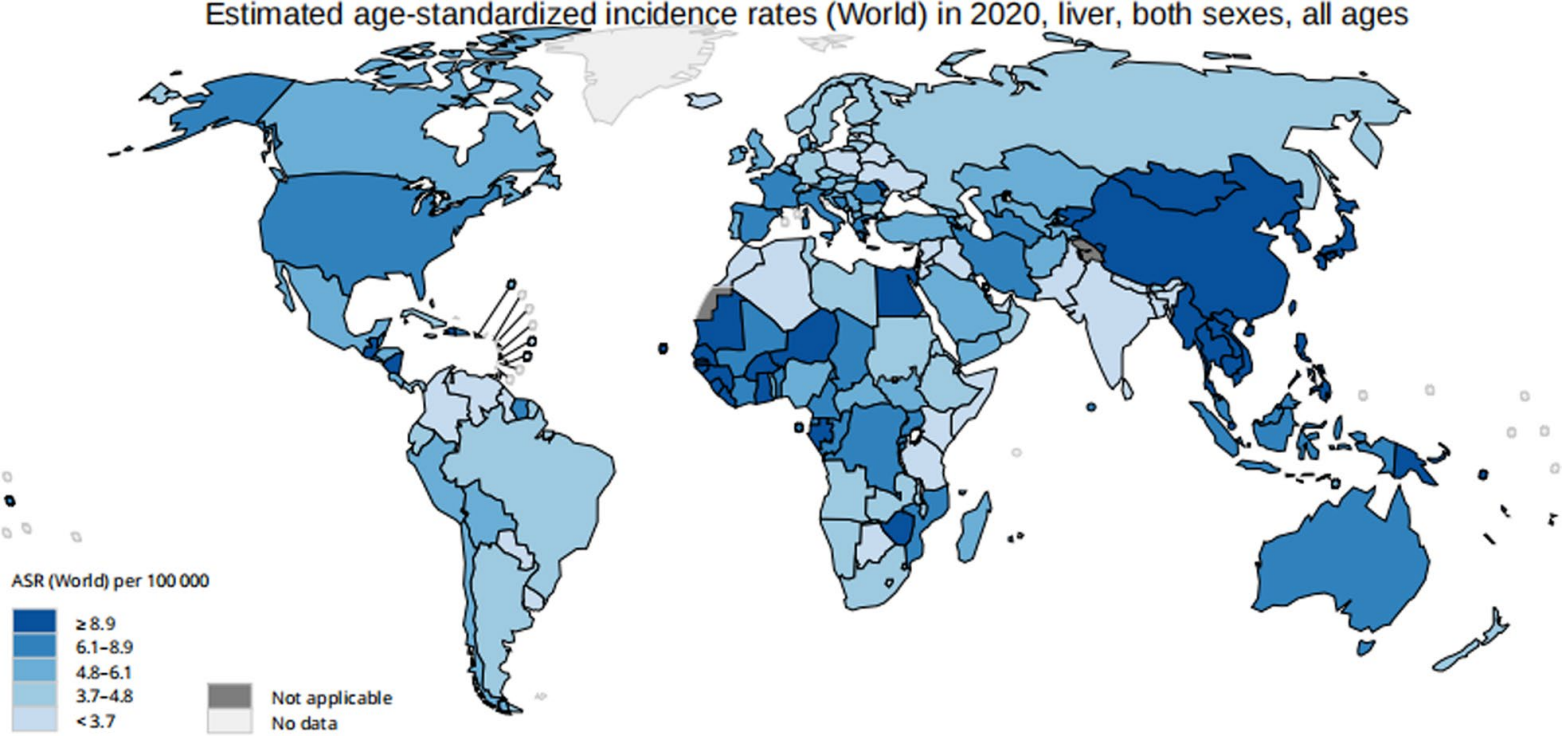
Global liver cancer distribution in 2020 (http://gco.iarc.fr/today)

On the basis of Global Cancer 2020 statistics, the new incidence of liver cancer was approximately 905,677 mainly occurring in Asia (Fig. 1), which is one of the five most prevalent types of cancer in Asia [1], It is also one of the causes of cancer death worldwide [2]. Additionally, malignant tumors of the liver are also one of the main factors of death from cancer in men [1], of which hepatocellular carcinoma (HCC) accounts for approximately 80% of global malignant liver tumor cases [2]. Viral infections leading to hepatitis B and C, aflatoxin infection, drinking, genetic susceptibility, as well as metabolic syndrome are recognized risk factors, with unidentified modifiable risk factors[3]. Recent researches have suggested that innate immunity has attracted much attention in the field of cancer, such as natural killer(NK) cells [4].

One of the essential components of the congenital immunity system is NK cells, which are capable of recognizing and killing virus-infected host cells [5]. In recent decades, NK cells have been confirmed in numerous studies to kill malignant tumor cells by recruiting to the position of tumorigenesis under the action of chemotactic factors released by other immune cells, thus inhibiting neoplasm development, invasion and distant metastasis [6],such as: lung cancer [7], hematological tumors [8, 9], glioblastoma [10], pancreatic cancer [11], breast cancer [12] and so on. Recent studies have also claimed that NK cells are extremely correlated with HCC, with a decrease in NK cells numbers leading to rapid growth of liver cancer cells and that the poor prognosis of HCC patients is closely related to cellular dysfunction or failure [13–15].Thus, it appears that NK cells play the pivotal role in HCC [16]. However, due to some potential biases in studies, such as confounding factors or reverse causation, whether there is a causal correlation between NK cell-associated immune traits and development of HCC remains to be further confirmed and systematically studied.

Mendelian randomization (MR)is a statistical method that uses genetic variation to assess causality. It leverages the natural random distribution of genetic variation in a population to mimic the characteristics of a randomized controlled trial and infer the causal impact of one factor on another [17]. effectively avoids the effects of confounding factors and reverse causality in observational studies by using genetic mutations as an instrumental variable for exposure to infer a causality of research results [18]. Presently, the causal relationship between HCC and potential risk factors has been extensively explored, including alcohol consumption [19] dysbacteriosis of the gut flora [20], telomere length [21], and metabolic syndrome [22]. In this paper, the causality between NK cell relevant immune traits and HCC was assessed by two-sample MR study using aggregated data from the newest and largest GWAS meta-analyses.

## Material and methods

### Data sources for exposure and outcomes

**Fig. 2:**
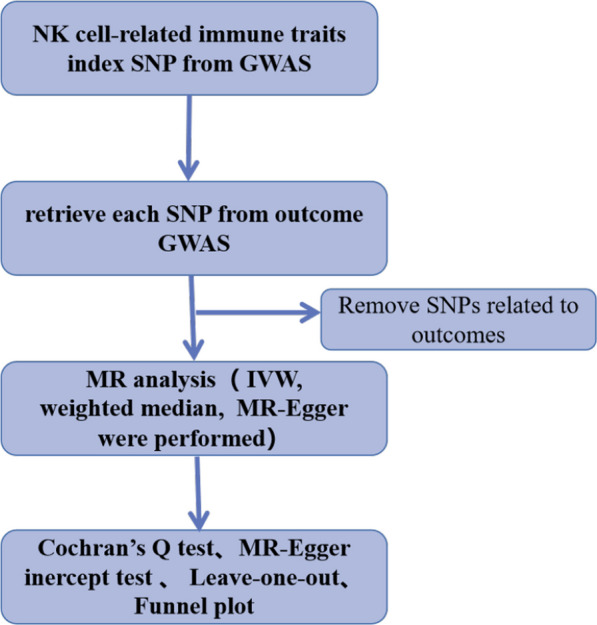
Mendelian randomization(MR) flowchart reveals causal relationship between NK cell-related immune traits and HCC

The steps of our study are presented in Fig. 2 below. Genetic tools which were derived from pooled data from the newest GWAS meta-analyses of NK cell-related immune traits in patients of European blood lineage (https://gwas.mrcieu.ac.uk/) were employed to reveal the causality between NK cell-related immunological traits and HCC. Moreover, nine independent association results were obtained in a large GWAS summary count of NK cell-related immune traits, including NK cells and CD45, SSC-A, CD16-CD56 on NK cells, CD45 on HLA-DR NK cells, CD8 + NKT cells, HLA DR NK/NK + cells, NK cells/CD3-lymphocytes, and HLA DR + NK cells/CD3-lymphocytes. "SSC-A" is one of the parameters used to assess cell granularity in flow cytometry [23]. In the case of NK cells, it is their granularity that is measured, which allows NK cells to be easily distinguished from other immune cells [24]. The GWAS summary statistics for HCC (ieu-b-4953) from the UK Biobanking Consortium contained 372,184 female and male subjects of European ancestry, with 168 cases and 372,016 controls.

### Selection of tool variables

Selection criteria for instrumental variables (IV): (1) When the significance threshold of GWAS was set to *p* < 5 × 10^–8^, the number of single nucleotide polymorphisms (SNPs) was less than three. Therefore, to ensure that there are enough SNPs for analysis, we adjusted the threshold to p < 5 × 10^–6^ [25], and chose SNPs reliably correlated with NK cell-associated immune traits as IVs;, (2) SNPs should satisfy the chain imbalance (r2 < 0.001, kb = 10,000); (3) SNPs containing A/T or G/C alleles which were excluded were called palindromic SNPs; (4) SNPs strongly correlated with outcomes were removed; (5) Statistical strength setting: retain strongly correlated IVs with F statistics > 10 for individual SNPs, and remove weakly correlated IVs with F < 10 [26].

### MR analysis and sensitive analysis

As aforementioned, the causality between NK cell-related immune traits and HCC was inferred by MR analysis. Inverse variance weighted (IVW) method was primarily utilized for MR analysis owing to its advantages of high statistical efficiency and providing more reliable results [27], In contrast, the MR-Egger method and WM could be used as complements to IVW estimates with more robustness, although they were less statistically efficient [28]. Since the results of MR analysis might be subject to pleiotropic bias, the next step was to test the reliability of the obtained results by means of sensitivity analysis. The causal estimates for each tool were visualized through visual funnel diagrams and Egger intercepts were implemented to assess multi-effectiveness [29]. Additionally, the presence of outliers highly influencing the effect value was examined based on leave-one-out analysis [30]. Cochran's Q was used to detect heterogeneity in the IVW analysis, with *p* < 0.05 indicating the presence of heterogeneity in the genetic variation instrument. If heterogeneity existed, the MR-PRESSO test was conducted to single out outliers to make suitable adjustments. However, the presence of heterogeneity could be acceptable when random-effects IVW was used as the primary outcome [31].

### Statistical analysis

All analysis were implemented in an R surrounding (version 4.2.3),and R pack TwoSampleMR (version 0.5.6) [32].

## Results

**Fig. 3:**
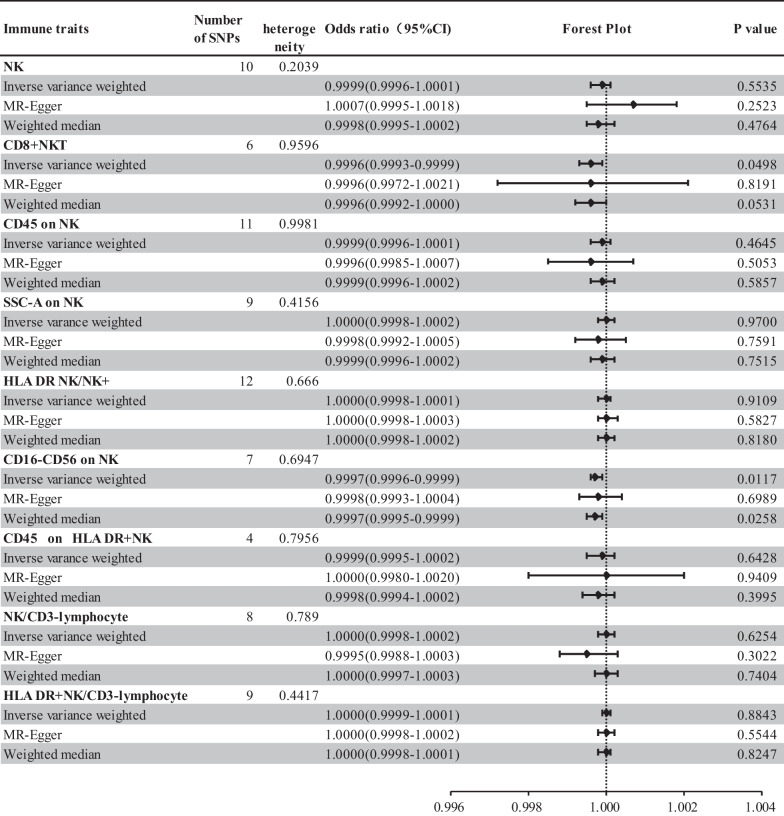
Estimation of NK cell-related immune traits with hepatocellular carcinoma(HCC) causal effect forest plot

There were 76 SNPs linked with NK cell relevant immune traits in this study (Additional file 1: Table 1 and Additional file 2),Interestingly, CD8^+^NKT cells and CD16^−^CD56 on NK cells exhibited negative correlation with hepatocellular carcinoma, suggesting a protective effect of CD8^+^NKT cells and CD16^−^CD56 on NK cells on HCC. According to Fig. 3, specific forest plots for estimating each causal effect using different MR analysis methods were elucidated. Low expression of CD8^+^NKT cells were linked with a higher risk of HCC in IVW analysis (OR 0.9996;95%CI 0.9993–0.9999; *P* = 0.0489), but MR-Egger analysis (OR0.9996;95%CI0.9972–1.0021; *P* = 0.8191) and WM analysis (OR0.9996; 95%CI0.9992–1.0000; *P* = 0.0531) showed no causal effect on HCC. The results, in IVW analysis (OR0.9997;95%CI 0.9996–0.9999;* P* = 0.0117) and WM analysis (OR0.9997; 95%CI0.9995–0.9999; *P* = 0.0258), suggested that elevated CD16-CD56 on NK cells was related with a reduced hazard of HCC development. Although the results of MR-Egger analysis turned out to be non-supportive for the causal correlation on HCC (OR0.9998;95%CI 0.9993–1.0004; *P* = 0.6989), IVW was considered as the primary outcome because of its high statistical efficiency and reliable results [31].

**Fig. 4:**
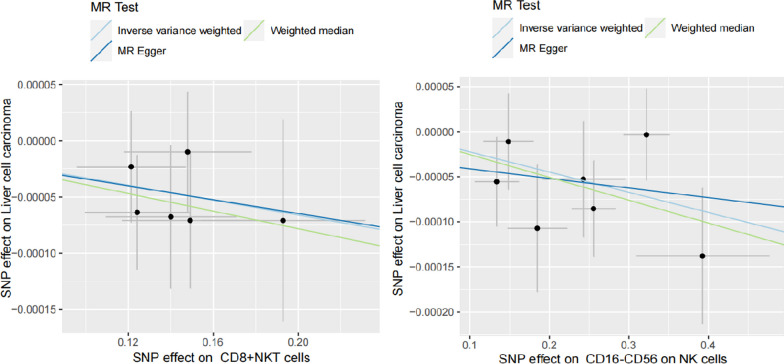
The effect of the same SNP on exposure is placed on the horizontal axis and the effect on outcome is placed on the vertical axis

**Fig. 5:**
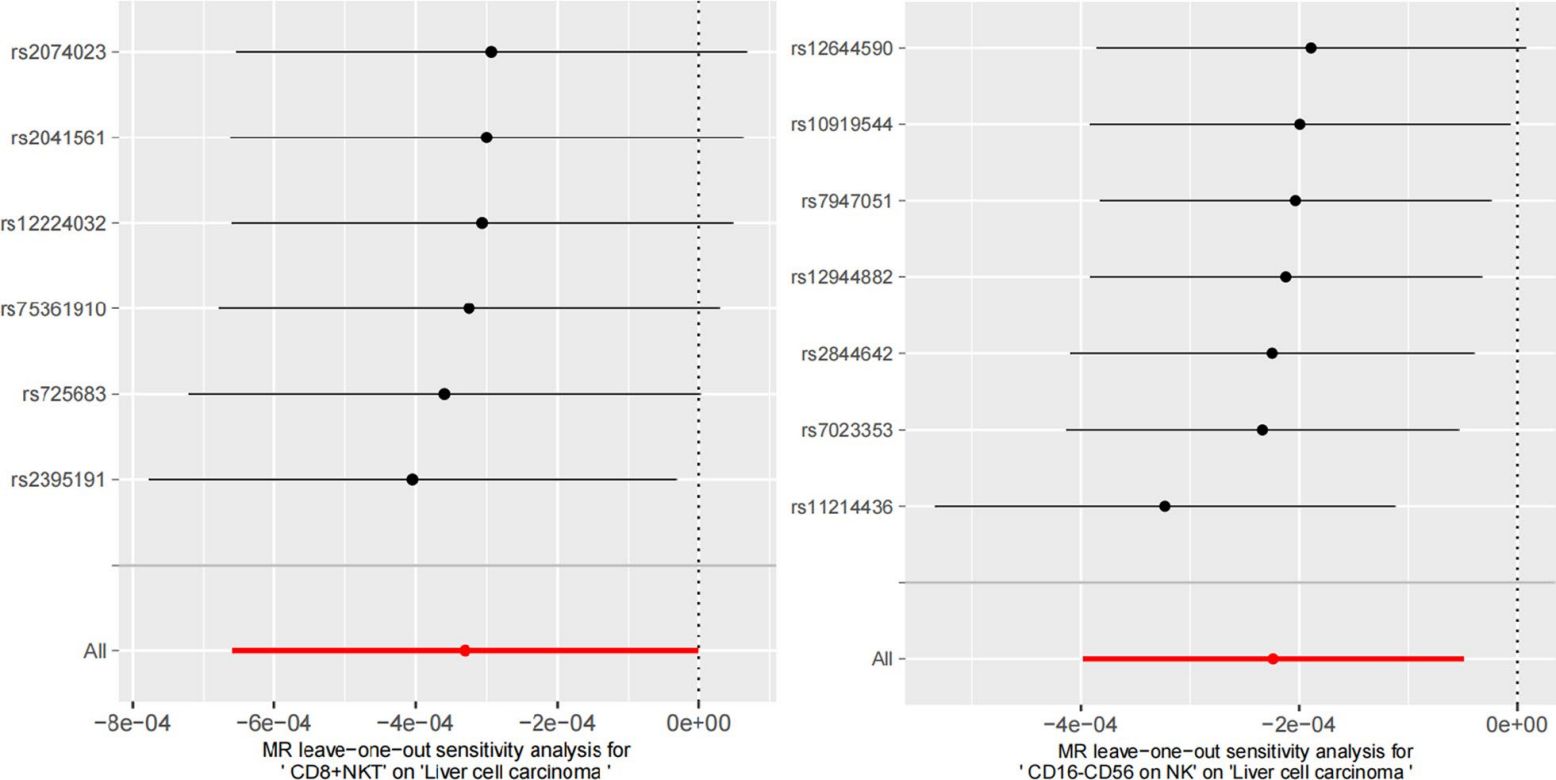
Forest plots for the Mendelian randomization (MR) leave-one-out sensitivity analysis of single SNP on HCC

For prominence and nominal prominence estimates (IVW *P* < 0.05), intercept from Egger showed the non-existence of pleiotropy and confounding factors in the study (Fig. 4). Visual funnel diagrams revealed tool variables in a roughly funnel shape, disregarding directional pleiotropy (Additional file 1: Figure S1). Leave-one-out analysis confirmed unobserved outliers that imposed great impacts on the effect value (Fig. 5). Besides, it was found that the causal effect estimates were not biased by individual instrumental variables, indicating that the estimates were reliable and consistent with the assumptions (Fig. 5). According to Cochran's Q test, there was insufficient evidence to support heterogeneity (Fig. 3).

## Discussion

Our research mainly relied on MR analysis to decide on causality between NK cell-related immune traits and HCC on the basis of systematic and scientific analysis. The findings indicated a causal relationship between CD8^+^ NKT cells and CD16^−^CD56 on NK cells and reduced risk of HCC, suggesting that they were protective factors for HCC, supporting the earlier findings that provided a solid foundation for our study and demonstrated the significance and reliability of our results [33–36].

NK cells play a major character in tumor development as powerful innate immune lymphocytes that mediate the immune surveillance and clearance of tumor cells [8]. When tumor cells are shed into the peripheral circulation as "seeds" to metastasize to distant sites, NK cells serve as a role of monitoring the escape of circulating tumor cells through immune detection point molecules such as HLA-E:CD94-NKG2A, thus inhibiting the metastasis of cancer cells through the peripheral circulation [37, 38]. Additionally, NK cells are associated with the coordinated attack of tumor cells by immune cells in the tumor microenvironment and impede metastasis [39]. Currently, HCC remains one of the most universal and deadly cancers throughout the world, with a high morbidity and mortality rates, especially among men [40]. Evidence supported that the proportion of NK cells in the peripheral circulation was remarkably reduced in HCC patients compared to healthy volunteers, resulting in poor prognosis [41]. Zhang PF and his colleagues found that NK cell depletion and impaired function were caused by the HCC-derived exosome circUHRF1 through upregulation of TIM-3 expression, thereby inhibiting the production of IFN-γ and TNF-α by NK cells, and ultimately leading to rapid growth and spread of cancer cells [16, 42].Therefore, NK cells can be regarded as potential targets in the areas of HCC therapy.

In our findings, CD16^−^CD56 on NK cells was a protective factor for HCC. Previously, there were two subpopulations of NK cells in blood other than bone marrow, CD16CD56^+bright/dim^ NK cells and CD16^−^CD56^+bright/dim^ NK cells, based on CD56 surface density (bright, dim) and the presence or absence of CD16 [43, 44]. In the normal population, the former predominates, accounting for over 90% of peripheral blood NK cells, which plays a major role in alleviating cytotoxicity, while the latter accounts for a smaller proportion of peripheral blood NK, around 10%, mainly secreting cytokines to participate in immune regulation and inflammatory responses [45, 46]. Actually, periphery CD16-CD56 + bright/dim NK cells do not start with anti-tumor activity, but can rapidly proliferate and transform into CD16CD56^+bright^ NK cells after stimulation by cytokines (IL-2, IL-12 and IL-15) to acquire anti-tumor cytotoxic capacity [35]. Chen X and his team found that patients with an advanced stage of HCC treated with the combination of sintilimab and anlotinib had a higher proportion of CD16CD56 NK cells in the periphery with better anti-HCC outcomes and prognostic effects [47].

In addition, CD8^+^ NKT cells were also discovered to be tutelar elements for HCC, with a reduced risk of HCC development. NKT cells are specialized immune cells that can convey both αβ-TCR and receptors associated with NK cells located on the cytoplasmic membrane [48, 49]. Traditionally, depending on whether or not CD4/CD8 is present, it is divided into two subgroups, namely CD4^+^NKT and CD8^+^NKT [50], CD8^+^NKT cells against malignancy are associated with a Th1-biased response and homologous CD3 T cells, while CD4^+^NKT are mainly associated with immune regulation [51]. NKT cells activate the release of IFN-γ in HCC mainly through exogenous glycolipids (a-GalCer) to exert anti-tumor effects, and IL-4 can also activate N KT cells [34, 52]. In the TRAMP mouse model, TRAMP mice lacking NKT cells were more likely to experience increased tumor growth and metastasis, resulting in increased mortality in TRAMP mice [53]. Moreover, the proportion of NKT cells is significantly decreased in patients with malignant tumors and is significantly associated with patient prognosis [54, 55]. Thus CD8^+^NKT cells indicate negative correlation with hepatocellular carcinoma, supporting a protective effect of CD8^+^NKT cells on HCC. These findings will provide new strategies for the treatment of HCC.

In this paper, MR study was first executed to explore causality between NK cells associated immune traits and HCC. However, our study also has a number of limitations. Firstly, data on NK cells-related immune traits for our GWAS meta-analysis were primarily collected from the European population. Therefore, whether our results are consistent with that of research on non-European populations still needs to be comprehensively considered for future MR analyses in both European and non-European populations, in order to enhance the generalizability and rigor of the study. Secondly, in conducting the MR analyses, we utilized retrospective data. While the findings suggest a potential causal relationship, the evidence is insufficient to establish definitive proof. Further studies, such as randomized case–control trials, will be necessary to confirm causality. Thirdly, in terms of the selection of instrumental variables, *p* < 5 × 10^–6^ was chosen for the GWAS significance threshold. Although previous studies also used it [25],there might be some weak instrumental variables. For this reason, the F value of each SNP statistic was calculated, with the F value greater than 10, regardless of the existence of a weak correlation (Additional file 1: Table 2).

## Conclusions

As mentioned MR study suggests a causal relationship between NK cell-related immune traits and HCC. CD16^−^CD56 on NK cells and CD8^+^ NKT cells might act as protective factors against HCC and high expression might be related with a lower risk of HCC.

### Supplementary Information

**Additional file 1:** Detailed information on instrumental variables in MR analysis.

**Additional file 2:** Exposure and outcome harmonize data.

## Data Availability

The data for this study can be found online in the IEU database. https://gwas.mrcieu.ac.uk/datasets/ Available as an open database.

## Abbreviations

HCC: Hepatocellular carcinoma
MR: Mendelian Randomization
GWAS: Genome-wide association studies
SNPs: Selection of single nucleotide polymorphisms
IVW: Inverse variance weighted
NK: Natural killer
