# Supplementary material for: The causality between CD8+NKT cells and CD16−CD56 on NK cells with hepatocellular carcinoma: a Mendelian randomization study
Source: Infect Agent Cancer. 2024 Jan 20;19:3. doi: 10.1186/s13027-024-00565-8 (PMC10799464; doi:10.1186/s13027-024-00565-8)
Supplement: Supplementary file 1 — Additional file 1: Detailed information on instrumental variables in MR analysis. [file 13027_2024_565_MOESM1_ESM.docx]

**Supplementary Materials**

CD8^+^NKT cells and CD16^-^CD56 on NK cells are causally related with hepatocellular carcinoma: a Mendelian randomization study

| **Supplementary Table 1. Detailed information of instrumental variables used in MR analyses** | | | | | | | | | | |
| --- | --- | --- | --- | --- | --- | --- | --- | --- | --- | --- |
| Trait | Immune traits | SNP | Effect allele | Other allele | Exposure | | | Outcome (HCC traits) | | |
|  |  |  |  |  | Beta | SE | P-value | Beta | SE | P-value |
| HCC | NK | rs1257331 | T | C | 0.1177 | 0.02464 | 1.83E-06 | -4.12E-05 | 5.31E-05 | 0.44 |
| HCC | NK | rs184712 | T | C | -0.188 | 0.03968 | 2.23E-06 | 9.06E-05 | 6.30E-05 | 0.15 |
| HCC | NK | rs34341378 | T | A | 0.1561 | 0.0322 | 1.30E-06 | -2.53E-05 | 5.92E-05 | 0.67 |
| HCC | NK | rs3789102 | T | C | 0.1119 | 0.02443 | 4.76E-06 | -1.93E-05 | 4.94E-05 | 0.7 |
| HCC | NK | rs55971447 | T | C | -0.1314 | 0.02854 | 4.29E-06 | -7.09E-05 | 7.61E-05 | 0.35 |
| HCC | NK | rs59536859 | G | A | -0.1659 | 0.03479 | 1.92E-06 | 0.000104326 | 6.97E-05 | 0.13 |
| HCC | NK | rs597808 | G | A | -0.1204 | 0.02575 | 3.05E-06 | 8.48E-05 | 4.94E-05 | 0.09 |
| HCC | NK | rs7216096 | A | G | 0.2022 | 0.03265 | 6.55E-10 | -1.54E-06 | 6.55E-05 | 0.98 |
| HCC | NK | rs935320 | T | A | -0.2003 | 0.04335 | 3.96E-06 | -0.000117439 | 9.14E-05 | 0.2 |
| HCC | NK | rs9916257 | T | G | -0.2005 | 0.02398 | 8.82E-17 | -6.90E-05 | 4.97E-05 | 0.16 |
| HCC | CD8+NKT | rs12224032 | G | C | 0.14 | 0.03055 | 4.76E-06 | -6.77E-05 | 6.35E-05 | 0.29 |
| HCC | CD8+NKT | rs2041561 | C | G | 0.1491 | 0.0321 | 3.52E-06 | -7.09E-05 | 6.00E-05 | 0.24 |
| HCC | CD8+NKT | rs2074023 | T | C | -0.1242 | 0.02469 | 5.06E-07 | 6.37E-05 | 5.08E-05 | 0.21 |
| HCC | CD8+NKT | rs2395191 | C | T | 0.1479 | 0.0299 | 7.87E-07 | -9.93E-06 | 5.30E-05 | 0.85 |
| HCC | CD8+NKT | rs725683 | T | C | -0.1213 | 0.02557 | 2.18E-06 | 2.31E-05 | 4.93E-05 | 0.64 |
| HCC | CD8+NKT | rs75361910 | T | C | -0.1928 | 0.03853 | 5.85E-07 | 7.09E-05 | 8.94E-05 | 0.43 |
| HCC | CD45 on NK | rs10882100 | G | C | 0.1317 | 0.02741 | 1.63E-06 | 2.98E-05 | 4.93E-05 | 0.55 |
| HCC | CD45 on NK | rs1182654 | T | C | 0.1297 | 0.02755 | 2.61E-06 | 3.77E-06 | 5.06E-05 | 0.94 |
| HCC | CD45 on NK | rs12593800 | C | T | -0.1265 | 0.02713 | 3.22E-06 | -1.67E-05 | 5.30E-05 | 0.75 |
| HCC | CD45 on NK | rs12928699 | T | C | 0.2398 | 0.05098 | 2.67E-06 | -2.62E-05 | 8.84E-05 | 0.77 |
| HCC | CD45 on NK | rs2163548 | G | C | -0.1744 | 0.03788 | 4.35E-06 | 1.53E-05 | 7.00E-05 | 0.83 |
| HCC | CD45 on NK | rs2377027 | G | C | 0.159 | 0.02795 | 1.39E-08 | 6.70E-06 | 4.93E-05 | 0.89 |
| HCC | CD45 on NK | rs4327090 | G | A | 0.1656 | 0.0352 | 2.63E-06 | 1.00E-06 | 6.25E-05 | 0.99 |
| HCC | CD45 on NK | rs6721978 | C | T | 0.2237 | 0.02785 | 1.34E-15 | -4.17E-05 | 5.84E-05 | 0.48 |
| HCC | CD45 on NK | rs72677702 | G | T | 0.1431 | 0.03058 | 3.02E-06 | -8.87E-06 | 5.53E-05 | 0.87 |
| HCC | CD45 on NK | rs72756114 | A | C | -0.1318 | 0.02873 | 4.69E-06 | 7.44E-06 | 5.38E-05 | 0.89 |
| HCC | CD45 on NK | rs751514 | C | T | -0.1571 | 0.03173 | 7.80E-07 | 5.68E-05 | 6.52E-05 | 0.38 |
| HCC | SSC-A on NK | rs1006368 | T | C | 0.4144 | 0.04052 | 3.61E-24 | -7.15E-05 | 7.86E-05 | 0.36 |
| HCC | SSC-A on NK | rs10512469 | T | C | -0.1684 | 0.03658 | 4.31E-06 | 4.54E-06 | 6.52E-05 | 0.94 |
| HCC | SSC-A on NK | rs11703193 | A | G | -0.1793 | 0.03727 | 1.57E-06 | -8.70E-05 | 6.66E-05 | 0.19 |
| HCC | SSC-A on NK | rs28691703 | C | T | 0.1706 | 0.03639 | 2.87E-06 | 2.20E-05 | 5.53E-05 | 0.69 |
| HCC | SSC-A on NK | rs6850820 | A | G | -0.1642 | 0.03573 | 4.48E-06 | -6.60E-05 | 5.80E-05 | 0.26 |
| HCC | SSC-A on NK | rs6863338 | T | C | 0.2553 | 0.05018 | 3.85E-07 | 8.48E-05 | 7.80E-05 | 0.28 |
| HCC | SSC-A on NK | rs7575742 | G | A | 0.1383 | 0.02791 | 7.57E-07 | 1.87E-05 | 5.23E-05 | 0.72 |
| HCC | SSC-A on NK | rs767896 | T | C | 0.1637 | 0.03376 | 1.31E-06 | -5.48E-05 | 5.35E-05 | 0.31 |
| HCC | SSC-A on NK | rs9912354 | T | C | 0.1471 | 0.02719 | 6.80E-08 | -6.76E-05 | 4.99E-05 | 0.18 |
| HCC | HLA DR NK/NK+ | rs117338457 | T | G | 0.2936 | 0.06216 | 2.40E-06 | -2.66E-06 | 9.75E-05 | 0.98 |
| HCC | HLA DR NK/NK+ | rs1862176 | A | G | -0.14 | 0.02753 | 3.87E-07 | 6.28E-05 | 5.37E-05 | 0.24 |
| HCC | HLA DR NK/NK+ | rs4656984 | C | T | 0.1389 | 0.02628 | 1.32E-07 | 5.40E-05 | 5.08E-05 | 0.29 |
| HCC | HLA DR NK/NK+ | rs6833916 | T | G | 0.1416 | 0.03037 | 3.26E-06 | 5.99E-05 | 6.48E-05 | 0.35 |
| HCC | HLA DR NK/NK+ | rs71632989 | G | T | -0.6848 | 0.02772 | 1.30E-124 | -6.78E-05 | 7.61E-05 | 0.37 |
| HCC | HLA DR NK/NK+ | rs7180804 | A | G | -0.2525 | 0.03019 | 8.74E-17 | 4.84E-06 | 5.64E-05 | 0.93 |
| HCC | HLA DR NK/NK+ | rs72685367 | G | C | 0.3669 | 0.07597 | 1.43E-06 | -4.21E-05 | 9.16E-05 | 0.65 |
| HCC | HLA DR NK/NK+ | rs72770017 | A | G | -0.1367 | 0.02968 | 4.26E-06 | -5.68E-05 | 5.65E-05 | 0.32 |
| HCC | HLA DR NK/NK+ | rs74341264 | A | G | 0.3328 | 0.03867 | 1.13E-17 | -6.27E-05 | 9.86E-05 | 0.52 |
| HCC | HLA DR NK/NK+ | rs80171191 | T | C | -0.1619 | 0.03433 | 2.49E-06 | 7.89E-05 | 9.09E-05 | 0.39 |
| HCC | HLA DR NK/NK+ | rs865239 | G | A | -0.1371 | 0.02509 | 4.97E-08 | 1.32E-05 | 4.94E-05 | 0.79 |
| HCC | HLA DR NK/NK+ | rs9916257 | T | G | 0.1457 | 0.02506 | 6.72E-09 | -6.90E-05 | 4.97E-05 | 0.16 |
| HCC | CD16-CD56 on NK | rs10919544 | C | T | -0.2555 | 0.02745 | 2.40E-20 | 8.55E-05 | 5.33E-05 | 0.11 |
| HCC | CD16-CD56 on NK | rs11214436 | T | G | 0.3221 | 0.02878 | 1.59E-28 | -3.36E-06 | 5.08E-05 | 0.95 |
| HCC | CD16-CD56 on NK | rs12644590 | G | A | 0.3926 | 0.08376 | 2.90E-06 | -0.000137859 | 7.53E-05 | 0.07 |
| HCC | CD16-CD56 on NK | rs12944882 | C | T | -0.1336 | 0.02747 | 1.22E-06 | 5.55E-05 | 4.95E-05 | 0.26 |
| HCC | CD16-CD56 on NK | rs2844642 | A | G | -0.2426 | 0.05192 | 3.10E-06 | 5.27E-05 | 6.39E-05 | 0.41 |
| HCC | CD16-CD56 on NK | rs7023353 | C | T | 0.1483 | 0.0313 | 2.24E-06 | -1.10E-05 | 5.33E-05 | 0.84 |
| HCC | CD16-CD56 on NK | rs7947051 | G | A | 0.1847 | 0.03723 | 7.42E-07 | -0.000107095 | 7.04E-05 | 0.13 |
| HCC | CD45 on HLA DR+ NK | rs12772032 | T | A | -0.1338 | 0.02881 | 3.57E-06 | 3.46E-05 | 5.29E-05 | 0.51 |
| HCC | CD45 on HLA DR+ NK | rs1860345 | C | T | -0.1714 | 0.03604 | 2.07E-06 | -3.18E-05 | 5.58E-05 | 0.57 |
| HCC | CD45 on HLA DR+ NK | rs2495272 | G | A | 0.1636 | 0.03453 | 2.26E-06 | -3.17E-05 | 6.52E-05 | 0.63 |
| HCC | CD45 on HLA DR+ NK | rs7698455 | T | A | 0.2256 | 0.04849 | 3.41E-06 | -3.94E-05 | 7.87E-05 | 0.62 |
| HCC | NK/CD3- lymphocyte | rs12087279 | T | C | -0.1667 | 0.03634 | 4.64E-06 | -9.02E-06 | 6.84E-05 | 0.9 |
| HCC | NK/CD3- lymphocyte | rs17823772 | T | C | -0.2182 | 0.04488 | 1.21E-06 | 2.41E-05 | 7.66E-05 | 0.75 |
| HCC | NK/CD3- lymphocyte | rs281407 | A | G | 0.1175 | 0.02521 | 3.24E-06 | 1.72E-05 | 5.31E-05 | 0.75 |
| HCC | NK/CD3- lymphocyte | rs55971447 | T | C | -0.1488 | 0.02845 | 1.77E-07 | -7.09E-05 | 7.61E-05 | 0.35 |
| HCC | NK/CD3- lymphocyte | rs7218011 | G | A | 0.1822 | 0.03225 | 1.74E-08 | -3.39E-06 | 6.54E-05 | 0.96 |
| HCC | NK/CD3- lymphocyte | rs73174907 | A | T | -0.1949 | 0.03808 | 3.23E-07 | -2.49E-05 | 6.25E-05 | 0.69 |
| HCC | NK/CD3- lymphocyte | rs73520180 | C | T | -0.3941 | 0.08347 | 2.44E-06 | 9.10E-05 | 9.21E-05 | 0.32 |
| HCC | NK/CD3- lymphocyte | rs9916257 | T | G | -0.1909 | 0.02375 | 1.21E-15 | -6.90E-05 | 4.97E-05 | 0.16 |
| HCC | HLA DR+NK /CD3- lymphocyte | rs1609454 | G | A | -0.1188 | 0.02587 | 4.50E-06 | -1.35E-05 | 6.01E-05 | 0.82 |
| HCC | HLA DR+NK /CD3- lymphocyte | rs1862176 | A | G | -0.1461 | 0.02698 | 6.50E-08 | 6.28E-05 | 5.37E-05 | 0.24 |
| HCC | HLA DR+NK /CD3- lymphocyte | rs2880640 | C | T | -0.1254 | 0.02609 | 1.59E-06 | -1.07E-05 | 5.07E-05 | 0.83 |
| HCC | HLA DR+NK /CD3- lymphocyte | rs28816203 | A | G | 0.1225 | 0.02549 | 1.61E-06 | -0.000102291 | 5.48E-05 | 0.06 |
| HCC | HLA DR+NK /CD3- lymphocyte | rs62028061 | C | T | -0.1225 | 0.0259 | 2.35E-06 | -3.10E-05 | 5.95E-05 | 0.60 |
| HCC | HLA DR+NK /CD3- lymphocyte | rs71632979 | G | A | -0.7124 | 0.02686 | 9.98E-142 | -6.94E-05 | 7.61E-05 | 0.36 |
| HCC | HLA DR+NK /CD3- lymphocyte | rs72685367 | G | C | 0.3452 | 0.0745 | 3.73E-06 | -4.21E-05 | 9.16E-05 | 0.65 |
| HCC | HLA DR+NK /CD3- lymphocyte | rs74341264 | A | G | 0.3424 | 0.03788 | 2.53E-19 | -6.27E-05 | 9.86E-05 | 0.52 |
| HCC | HLA DR+NK /CD3- lymphocyte | rs8025803 | T | A | -0.2729 | 0.0334 | 4.22E-16 | 1.28E-05 | 5.61E-05 | 0.82 |
|  |  |  |  |  |  |  |  |  |  |  |
| Notes: HCC:Hepatocellular carcinoma. Beta: Estimate coefficient; P-value: P-value from GWAS ; SE: standard error of coefficient estimate. | | | | | | | | | | |
|  |  |  |  |  |  |  |  |  |  |  |

| **Supplementary Table 2. Detailed information of exposure used in MR analyses** | | | | | | | |  |  |
| --- | --- | --- | --- | --- | --- | --- | --- | --- | --- |
| Trait | Immune traits | SNP | Effect allele | Other allele | Exposure | | | | |
|  |  |  |  |  | Beta | MAF | SIZE | R2 | F statistics |
| HCC | NK | rs183053322 | T | C | -0.408 | 0.0354 | 3669 | 1.14E-02 | 42.17 |
| HCC | NK | rs6552764 | T | A | -0.1993 | 0.0767 | 3669 | 5.63E-03 | 20.75 |
| HCC | NK | rs77438219 | A | G | -1.131 | 0.0022 | 3669 | 5.62E-03 | 20.71 |
| HCC | NK | rs56311672 | T | A | -2.292 | 0.0005 | 3669 | 5.25E-03 | 19.36 |
| HCC | NK | rs11954223 | A | G | -1.682 | 0.001 | 3669 | 5.65E-03 | 20.85 |
| HCC | NK | rs10948071 | T | C | 0.1124 | 0.5327 | 3669 | 6.29E-03 | 23.21 |
| HCC | NK | rs2269705 | C | T | 0.1676 | 0.1956 | 3669 | 8.84E-03 | 32.70 |
| HCC | NK | rs10118740 | A | G | -0.2745 | 0.0412 | 3669 | 5.95E-03 | 21.96 |
| HCC | NK | rs2433423 | C | T | -0.4268 | 0.0177 | 3669 | 6.33E-03 | 23.38 |
| HCC | NK | rs10512469 | T | C | 0.249 | 0.1639 | 3669 | 1.70E-02 | 63.39 |
| HCC | NK | rs9916257 | T | G | -0.2323 | 0.5685 | 3669 | 2.65E-02 | 99.72 |
| HCC | NK | rs62130952 | A | G | -1.284 | 0.0016 | 3669 | 5.27E-03 | 19.42 |
| HCC | NK | rs281407 | A | G | 0.1157 | 0.3684 | 3669 | 6.23E-03 | 22.99 |
| HCC | NK | rs3787412 | A | C | -0.1923 | 0.0837 | 3669 | 5.67E-03 | 20.92 |
| HCC | NK | rs73174907 | A | T | -0.1857 | 0.1066 | 3669 | 6.57E-03 | 24.25 |
| HCC | CD8+NKT | rs34994982 | G | A | -0.1981 | 0.0994 | 3668 | 7.03E-03 | 25.94 |
| HCC | CD8+NKT | rs60553229 | A | G | -1.466 | 0.0014 | 3668 | 6.01E-03 | 22.16 |
| HCC | CD8+NKT | rs115889733 | A | G | 0.2125 | 0.0777 | 3668 | 6.47E-03 | 23.88 |
| HCC | CD8+NKT | rs2395191 | C | T | 0.1479 | 0.3721 | 3668 | 1.02E-02 | 37.86 |
| HCC | CD8+NKT | rs34575510 | T | C | 0.2652 | 0.0488 | 3668 | 6.53E-03 | 24.09 |
| HCC | CD8+NKT | rs2041561 | C | G | 0.1491 | 0.8213 | 3668 | 6.53E-03 | 24.08 |
| HCC | CD8+NKT | rs11544989 | G | A | 0.1222 | 0.6125 | 3668 | 7.09E-03 | 26.17 |
| HCC | CD8+NKT | rs725683 | T | C | -0.1213 | 0.5781 | 3668 | 7.18E-03 | 26.50 |
| HCC | CD8+NKT | rs12792298 | T | C | -0.3039 | 0.0345 | 3668 | 6.15E-03 | 22.70 |
| HCC | CD8+NKT | rs2074023 | T | C | -0.1242 | 0.4124 | 3668 | 7.48E-03 | 27.61 |
| HCC | CD8+NKT | rs78268116 | T | C | -0.3961 | 0.1095 | 3668 | 3.06E-02 | 115.71 |
| HCC | CD8+NKT | rs12224032 | G | C | 0.14 | 0.2087 | 3668 | 6.47E-03 | 23.89 |
| HCC | CD8+NKT | rs144156455 | C | T | 0.324 | 0.0361 | 3668 | 7.31E-03 | 26.98 |
| HCC | CD8+NKT | rs61944019 | A | G | -0.7638 | 0.0052 | 3668 | 6.04E-03 | 22.26 |
| HCC | CD8+NKT | rs117122100 | T | C | -0.4752 | 0.0142 | 3668 | 6.32E-03 | 23.32 |
| HCC | CD8+NKT | rs75361910 | T | C | -0.1928 | 0.117 | 3668 | 7.68E-03 | 28.37 |
| HCC | CD45 on NK | rs183053322 | T | C | 0.5102 | 0.0357 | 3113 | 1.79E-02 | 56.77 |
| HCC | CD45 on NK | rs75312377 | T | G | 0.5754 | 0.0165 | 3113 | 1.07E-02 | 33.79 |
| HCC | CD45 on NK | rs2377027 | G | C | 0.159 | 0.6002 | 3113 | 1.21E-02 | 38.21 |
| HCC | CD45 on NK | rs181102707 | T | G | 0.2952 | 0.0514 | 3113 | 8.50E-03 | 26.66 |
| HCC | CD45 on NK | rs2163548 | G | C | -0.1744 | 0.154 | 3113 | 7.93E-03 | 24.85 |
| HCC | CD45 on NK | rs6721978 | C | T | 0.2237 | 0.3466 | 3113 | 2.27E-02 | 72.15 |
| HCC | CD45 on NK | rs2331416 | C | T | -0.1294 | 0.5432 | 3113 | 8.31E-03 | 26.07 |
| HCC | CD45 on NK | rs72756114 | A | C | -0.1318 | 0.3155 | 3113 | 7.50E-03 | 23.52 |
| HCC | CD45 on NK | rs72502555 | G | C | -0.159 | 0.2621 | 3113 | 9.78E-03 | 30.72 |
| HCC | CD45 on NK | rs190229330 | T | G | -1.216 | 0.0029 | 3113 | 8.55E-03 | 26.83 |
| HCC | CD45 on NK | rs117491214 | T | C | 1.189 | 0.0022 | 3113 | 6.21E-03 | 19.43 |
| HCC | CD45 on NK | rs34664922 | C | A | -0.3393 | 0.0377 | 3113 | 8.35E-03 | 26.21 |
| HCC | CD45 on NK | rs10781534 | T | C | 0.1443 | 0.7613 | 3113 | 7.57E-03 | 23.72 |
| HCC | CD45 on NK | rs1182654 | T | C | 0.1297 | 0.4088 | 3113 | 8.13E-03 | 25.50 |
| HCC | CD45 on NK | rs10882100 | G | C | 0.1317 | 0.4998 | 3113 | 8.67E-03 | 27.22 |
| HCC | CD45 on NK | rs751514 | C | T | -0.1571 | 0.7695 | 3113 | 8.76E-03 | 27.48 |
| HCC | CD45 on NK | rs72677702 | G | T | 0.1431 | 0.2739 | 3113 | 8.15E-03 | 25.55 |
| HCC | CD45 on NK | rs146515193 | T | G | 0.8128 | 0.0064 | 3113 | 8.40E-03 | 26.36 |
| HCC | CD45 on NK | rs12593800 | C | T | -0.1265 | 0.4162 | 3113 | 7.78E-03 | 24.38 |
| HCC | CD45 on NK | rs12928699 | T | C | 0.2398 | 0.0765 | 3113 | 8.13E-03 | 25.48 |
| HCC | CD45 on NK | rs4327090 | G | A | 0.1656 | 0.2345 | 3113 | 9.85E-03 | 30.93 |
| HCC | CD45 on NK | rs77592206 | T | C | -0.3311 | 0.0337 | 3113 | 7.14E-03 | 22.37 |
| HCC | SSC-A on NK | rs7575742 | G | A | 0.1383 | 0.3696 | 3113 | 8.91E-03 | 27.98 |
| HCC | SSC-A on NK | rs6850820 | A | G | -0.1642 | 0.8275 | 3113 | 7.70E-03 | 24.13 |
| HCC | SSC-A on NK | rs77154024 | C | T | 1.113 | 0.0027 | 3113 | 6.67E-03 | 20.89 |
| HCC | SSC-A on NK | rs6863338 | T | C | 0.2553 | 0.084 | 3113 | 1.00E-02 | 31.52 |
| HCC | SSC-A on NK | rs79025422 | A | G | 0.5466 | 0.0148 | 3113 | 8.71E-03 | 27.34 |
| HCC | SSC-A on NK | rs767896 | T | C | 0.1637 | 0.2114 | 3113 | 8.93E-03 | 28.05 |
| HCC | SSC-A on NK | rs1006368 | T | C | 0.4144 | 0.1224 | 3113 | 3.69E-02 | 119.17 |
| HCC | SSC-A on NK | rs2198787 | C | A | -0.2652 | 0.9269 | 3113 | 9.53E-03 | 29.94 |
| HCC | SSC-A on NK | rs9912354 | T | C | 0.1471 | 0.5254 | 3113 | 1.08E-02 | 33.94 |
| HCC | SSC-A on NK | rs10512469 | T | C | -0.1684 | 0.1646 | 3113 | 7.80E-03 | 24.45 |
| HCC | SSC-A on NK | rs11081793 | G | A | -0.3945 | 0.0255 | 3113 | 7.73E-03 | 24.25 |
| HCC | SSC-A on NK | rs72901909 | G | T | -0.2893 | 0.058 | 3113 | 9.15E-03 | 28.71 |
| HCC | SSC-A on NK | rs28691703 | C | T | 0.1706 | 0.2127 | 3113 | 9.75E-03 | 30.62 |
| HCC | SSC-A on NK | rs11703193 | A | G | -0.1793 | 0.1597 | 3113 | 8.63E-03 | 27.08 |
| HCC | HLA DR NK/NK+ | rs865239 | G | A | -0.1371 | 0.5004 | 3596 | 9.40E-03 | 34.10 |
| HCC | HLA DR NK/NK+ | rs71632989 | G | T | -0.6848 | 0.2474 | 3596 | 1.75E-01 | 760.42 |
| HCC | HLA DR NK/NK+ | rs72685367 | G | C | 0.3669 | 0.0261 | 3596 | 6.84E-03 | 24.77 |
| HCC | HLA DR NK/NK+ | rs74341264 | A | G | 0.3328 | 0.1218 | 3596 | 2.37E-02 | 87.22 |
| HCC | HLA DR NK/NK+ | rs4656984 | C | T | 0.1389 | 0.6023 | 3596 | 9.24E-03 | 33.53 |
| HCC | HLA DR NK/NK+ | rs71424967 | T | C | 0.8583 | 0.0046 | 3596 | 6.75E-03 | 24.41 |
| HCC | HLA DR NK/NK+ | rs80171191 | T | C | -0.1619 | 0.1623 | 3596 | 7.13E-03 | 25.80 |
| HCC | HLA DR NK/NK+ | rs3135080 | G | A | -0.1222 | 0.642 | 3596 | 6.86E-03 | 24.84 |
| HCC | HLA DR NK/NK+ | rs6833916 | T | G | 0.1416 | 0.2045 | 3596 | 6.52E-03 | 23.60 |
| HCC | HLA DR NK/NK+ | rs1862176 | A | G | -0.14 | 0.6792 | 3596 | 8.54E-03 | 30.96 |
| HCC | HLA DR NK/NK+ | rs592776 | A | T | -0.6443 | 0.9914 | 3596 | 7.08E-03 | 25.62 |
| HCC | HLA DR NK/NK+ | rs17307458 | T | C | -0.5233 | 0.0128 | 3596 | 6.92E-03 | 25.05 |
| HCC | HLA DR NK/NK+ | rs145013190 | A | T | -0.4439 | 0.0209 | 3596 | 8.06E-03 | 29.22 |
| HCC | HLA DR NK/NK+ | rs117338457 | T | G | 0.2936 | 0.0449 | 3596 | 7.39E-03 | 26.77 |
| HCC | HLA DR NK/NK+ | rs7180804 | A | G | -0.2525 | 0.2162 | 3596 | 2.16E-02 | 79.37 |
| HCC | HLA DR NK/NK+ | rs72770017 | A | G | -0.1367 | 0.7729 | 3596 | 6.56E-03 | 23.73 |
| HCC | HLA DR NK/NK+ | rs9916257 | T | G | 0.1457 | 0.5698 | 3596 | 1.04E-02 | 37.80 |
| HCC | HLA DR NK/NK+ | rs148177889 | T | C | -0.5897 | 0.0096 | 3596 | 6.61E-03 | 23.92 |
| HCC | CD16-CD56 on NK | rs10919544 | C | T | -0.2555 | 0.5475 | 3113 | 3.23E-02 | 103.99 |
| HCC | CD16-CD56 on NK | rs139795227 | C | A | 0.3166 | 0.0496 | 3113 | 9.45E-03 | 29.68 |
| HCC | CD16-CD56 on NK | rs143549694 | G | A | 0.535 | 0.0161 | 3113 | 9.07E-03 | 28.47 |
| HCC | CD16-CD56 on NK | rs12644590 | G | A | 0.3926 | 0.0265 | 3113 | 7.95E-03 | 24.94 |
| HCC | CD16-CD56 on NK | rs75500437 | A | G | -0.2915 | 0.0504 | 3113 | 8.13E-03 | 25.51 |
| HCC | CD16-CD56 on NK | rs71607232 | A | G | 0.3965 | 0.0279 | 3113 | 8.53E-03 | 26.76 |
| HCC | CD16-CD56 on NK | rs2844642 | A | G | -0.2426 | 0.0996 | 3113 | 1.06E-02 | 33.19 |
| HCC | CD16-CD56 on NK | rs7023353 | C | T | 0.1483 | 0.262 | 3113 | 8.50E-03 | 26.69 |
| HCC | CD16-CD56 on NK | rs117655461 | T | C | -0.3041 | 0.0434 | 3113 | 7.68E-03 | 24.07 |
| HCC | CD16-CD56 on NK | rs111579151 | T | C | -0.5632 | 0.0323 | 3113 | 1.98E-02 | 62.94 |
| HCC | CD16-CD56 on NK | rs148849191 | T | C | -0.7822 | 0.0124 | 3113 | 1.50E-02 | 47.33 |
| HCC | CD16-CD56 on NK | rs17504675 | A | G | -0.4696 | 0.0308 | 3113 | 1.32E-02 | 41.51 |
| HCC | CD16-CD56 on NK | rs77291736 | T | C | -1.076 | 0.1086 | 3113 | 2.24E-01 | 898.84 |
| HCC | CD16-CD56 on NK | rs117533070 | T | C | 0.2856 | 0.0816 | 3113 | 1.22E-02 | 38.50 |
| HCC | CD16-CD56 on NK | rs182003559 | T | A | -0.9029 | 0.0072 | 3113 | 1.17E-02 | 36.69 |
| HCC | CD16-CD56 on NK | rs7947051 | G | A | 0.1847 | 0.1505 | 3113 | 8.72E-03 | 27.38 |
| HCC | CD16-CD56 on NK | rs11214436 | T | G | 0.3221 | 0.3055 | 3113 | 4.40E-02 | 143.27 |
| HCC | CD16-CD56 on NK | rs189384754 | G | T | 1.271 | 0.0026 | 3113 | 8.38E-03 | 26.29 |
| HCC | CD16-CD56 on NK | rs12944882 | C | T | -0.1336 | 0.4639 | 3113 | 8.88E-03 | 27.87 |
| HCC | CD16-CD56 on NK | rs151212014 | G | A | 0.6775 | 0.0082 | 3113 | 7.47E-03 | 23.40 |
| HCC | CD45 on HLA DR+ NK | rs2495272 | G | A | 0.1636 | 0.8179 | 2971 | 7.97E-03 | 23.86 |
| HCC | CD45 on HLA DR+ NK | rs7698455 | T | A | 0.2256 | 0.9137 | 2971 | 8.03E-03 | 24.02 |
| HCC | CD45 on HLA DR+ NK | rs142947331 | C | G | -0.319 | 0.0416 | 2971 | 8.11E-03 | 24.29 |
| HCC | CD45 on HLA DR+ NK | rs12772032 | T | A | -0.1338 | 0.3482 | 2971 | 8.13E-03 | 24.32 |
| HCC | CD45 on HLA DR+ NK | rs1860345 | C | T | -0.1714 | 0.1705 | 2971 | 8.31E-03 | 24.88 |
| HCC | CD45 on HLA DR+ NK | rs117580642 | G | C | -0.2462 | 0.0697 | 2971 | 7.86E-03 | 23.52 |
| HCC | NK/CD3- lymphocyte | rs12087279 | T | C | -0.1667 | 0.131 | 3669 | 6.33E-03 | 23.35 |
| HCC | NK/CD3- lymphocyte | rs183053322 | T | C | -0.4163 | 0.0354 | 3669 | 1.18E-02 | 43.92 |
| HCC | NK/CD3- lymphocyte | rs55971447 | T | C | -0.1488 | 0.237 | 3669 | 8.01E-03 | 29.60 |
| HCC | NK/CD3- lymphocyte | rs190518013 | T | A | -1.294 | 0.0019 | 3669 | 6.35E-03 | 23.44 |
| HCC | NK/CD3- lymphocyte | rs34588768 | T | C | 0.4242 | 0.017 | 3669 | 6.01E-03 | 22.19 |
| HCC | NK/CD3- lymphocyte | rs191368650 | T | G | 0.4173 | 0.0177 | 3669 | 6.06E-03 | 22.34 |
| HCC | NK/CD3- lymphocyte | rs11954223 | A | G | -2.093 | 0.001 | 3669 | 8.75E-03 | 32.38 |
| HCC | NK/CD3- lymphocyte | rs77438219 | A | G | -1.199 | 0.0022 | 3669 | 6.31E-03 | 23.29 |
| HCC | NK/CD3- lymphocyte | rs35483654 | T | C | -0.521 | 0.0123 | 3669 | 6.60E-03 | 24.35 |
| HCC | NK/CD3- lymphocyte | rs56311672 | T | A | -2.321 | 0.0005 | 3669 | 5.38E-03 | 19.85 |
| HCC | NK/CD3- lymphocyte | rs142527102 | C | T | -0.7471 | 0.0061 | 3669 | 6.77E-03 | 24.99 |
| HCC | NK/CD3- lymphocyte | rs73520180 | C | T | -0.3941 | 0.0217 | 3669 | 6.59E-03 | 24.34 |
| HCC | NK/CD3- lymphocyte | rs1915649 | T | C | -1.805 | 0.0014 | 3669 | 9.11E-03 | 33.71 |
| HCC | NK/CD3- lymphocyte | rs17823772 | T | C | -0.2182 | 0.0769 | 3669 | 6.76E-03 | 24.96 |
| HCC | NK/CD3- lymphocyte | rs2433423 | C | T | -0.4344 | 0.0177 | 3669 | 6.56E-03 | 24.22 |
| HCC | NK/CD3- lymphocyte | rs12874404 | G | A | -0.1456 | 0.319 | 3669 | 9.21E-03 | 34.09 |
| HCC | NK/CD3- lymphocyte | rs4239087 | C | T | -0.5865 | 0.9898 | 3669 | 6.95E-03 | 25.65 |
| HCC | NK/CD3- lymphocyte | rs9916257 | T | G | -0.1909 | 0.5685 | 3669 | 1.79E-02 | 66.76 |
| HCC | NK/CD3- lymphocyte | rs7218011 | G | A | 0.1822 | 0.1634 | 3669 | 9.08E-03 | 33.59 |
| HCC | NK/CD3- lymphocyte | rs111661128 | A | G | 0.3791 | 0.0232 | 3669 | 6.51E-03 | 24.04 |
| HCC | NK/CD3- lymphocyte | rs78468114 | T | C | -1.834 | 0.0008 | 3669 | 5.38E-03 | 19.83 |
| HCC | NK/CD3- lymphocyte | rs281407 | A | G | 0.1175 | 0.3684 | 3669 | 6.42E-03 | 23.71 |
| HCC | NK/CD3- lymphocyte | rs73174907 | A | T | -0.1949 | 0.1066 | 3669 | 7.24E-03 | 26.73 |
| HCC | HLA DR+NK /CD3- lymphocyte | rs72685367 | G | C | 0.3452 | 0.0261 | 3596 | 6.06E-03 | 21.91 |
| HCC | HLA DR+NK /CD3- lymphocyte | rs2880640 | C | T | -0.1254 | 0.3411 | 3596 | 7.07E-03 | 25.58 |
| HCC | HLA DR+NK /CD3- lymphocyte | rs74341264 | A | G | 0.3424 | 0.1218 | 3596 | 2.51E-02 | 92.46 |
| HCC | HLA DR+NK /CD3- lymphocyte | rs71632979 | G | A | -0.7124 | 0.2474 | 3596 | 1.89E-01 | 837.52 |
| HCC | HLA DR+NK /CD3- lymphocyte | rs71639910 | A | G | -0.2746 | 0.0685 | 3596 | 9.62E-03 | 34.92 |
| HCC | HLA DR+NK /CD3- lymphocyte | rs79676986 | C | A | 0.2654 | 0.0488 | 3596 | 6.54E-03 | 23.66 |
| HCC | HLA DR+NK /CD3- lymphocyte | rs3135080 | G | A | -0.1168 | 0.642 | 3596 | 6.27E-03 | 22.68 |
| HCC | HLA DR+NK /CD3- lymphocyte | rs1862176 | A | G | -0.1461 | 0.6792 | 3596 | 9.30E-03 | 33.74 |
| HCC | HLA DR+NK /CD3- lymphocyte | rs61745530 | T | G | -0.3596 | 0.0263 | 3596 | 6.62E-03 | 23.96 |
| HCC | HLA DR+NK /CD3- lymphocyte | rs145013190 | A | T | -0.4035 | 0.0209 | 3596 | 6.66E-03 | 24.11 |
| HCC | HLA DR+NK /CD3- lymphocyte | rs11595870 | A | G | 0.3054 | 0.0367 | 3596 | 6.59E-03 | 23.86 |
| HCC | HLA DR+NK /CD3- lymphocyte | rs8025803 | T | A | -0.2729 | 0.1783 | 3596 | 0.021822359 | 80.18 |
| HCC | HLA DR+NK /CD3- lymphocyte | rs62028061 | C | T | -0.1225 | 0.6379 | 3596 | 0.006932395 | 25.09 |
| HCC | HLA DR+NK /CD3- lymphocyte | rs28816203 | A | G | 0.1225 | 0.4053 | 3596 | 0.00723397 | 26.19 |
| HCC | HLA DR+NK /CD3- lymphocyte | rs150102419 | A | G | 0.6357 | 0.0074 | 3596 | 0.005936636 | 21.46 |
| HCC | HLA DR+NK /CD3- lymphocyte | rs1609454 | G | A | -0.1188 | 0.3407 | 3596 | 0.006340421 | 22.93 |
|  |  |  |  |  |  |  |  |  |  |


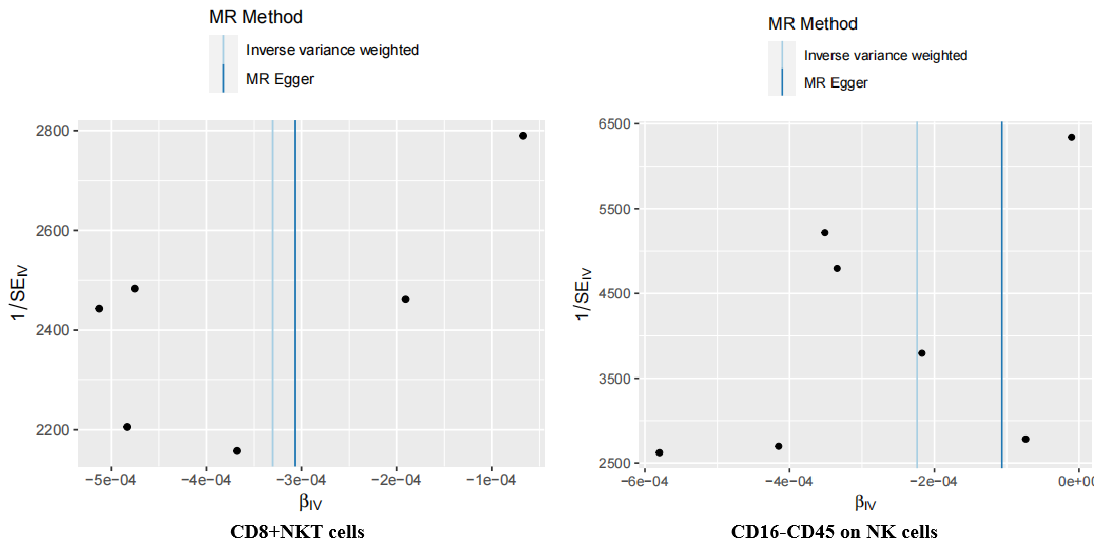


Figure S1Visualization of the funnel plots in the Mendelian randomization(MR) analysis
